# From minor loci to major players? Satellite DNA diversification in *Crepis sensu stricto*

**DOI:** 10.1007/s10577-025-09783-1

**Published:** 2025-11-05

**Authors:** Magdalena Senderowicz, Natalia Borowska-Żuchowska, Gülru Yücel, Teresa Nowak, Gbemisola Daini, Bozena Kolano

**Affiliations:** 1 Plant Cytogenetics and Molecular Biology Group, Institute of Biology, Biotechnology and Environmental Protection, Faculty of Natural Sciences, University of Silesia in Katowice, Katowice, 40-032 Poland; 2 Department of Agricultural Biotechnology, Faculty of Agriculture, Ondokuz Mayıs University, Samsun, 55139 Türkiye; 3 Botany and Nature Conservation Team, Institute of Biology, Biotechnology and Environmental Protection, Faculty of Natural Sciences, University of Silesia in Katowice, Katowice, Poland

**Keywords:** *Crepis*, Satellite sequences, Tandem repeats, Repeat homogenisation, Repetitive sequence evolution, Fluorescent in situ hybridisation, Southern hybridisation, Chromosomes

## Abstract

*Crepis sensu lato* (*s.l.*) comprises species with remarkable variation in genome size, chromosome number, and karyotype structure, making this genus a valuable model for studying genome and chromosome evolution. Studies in various plant systems have suggested that diversification and speciation may be accompanied by dynamic changes in the repetitive DNA fraction, including satellite DNAs (satDNAs). A complex approach consisting of molecular (cloning, dot-blot and Southern hybridisation), cytogenetic (fluorescent in situ hybridisation) and phylogenetic methods allowed assessing the chromosomal and genomic organisation of three satDNAs in 32 species from *Crepis s.l.*. In the present study, we posed a question of whether these satDNAs are specific to *C. capillaris*, supporting the “birth-and-death” hypothesis, or whether they are also present in related species, consistent with the “library hypothesis”. Each satDNA family exhibited different evolutionary trajectories. While pCcE9 amplification was specific to *C. capillaris*, supporting the “birth-and-death” hypothesis, two other analysed satDNAs were present in several related species, consistent with the “library hypothesis”. Notably, pCcD29 showed different genomic and chromosomal organisation among *C. capillaris* and the species from the *C. vesicaria* group, suggesting that the satDNA evolution model proposed by Ruiz-Ruano et al. for animal systems may also apply to *Crepis* species. The organisation of the pCcD29 family ranges from numerous small, poorly homogenised loci (*C. polymorpha*) through organisation in both shorter and longer arrays (*C. taraxacifolia*) to a few major, highly homogenised loci (*C. capillaris*), highlighting contrasting evolutionary pathways of satDNAs within closely related species.

**Supplementary Information:**

The online version contains supplementary material available at 10.1007/s10577-025-09783-1.

## Introduction

A significant portion (between 25 and 85%) of plant genomes consists of various types of repetitive DNA, which can be classified into two major categories based on their genomic organisation (Liu et al. 2019; Mehrotra and Goyal 2014). One category comprises elements with a dispersed organisation (mainly transposable elements), while the other consists of tandem repeats with adjacent monomers forming arrays (Garrido-Ramos 2017; Kubis et al. 1998; Macas et al. 2015; Pellicer et al. 2021). Tandem repetitive sequences include ribosomal RNA genes (rDNA) and a highly heterogeneous group of satellite DNAs (satDNAs; Garrido-Ramos 2017; Hemleben et al. 2007; Kubis et al. 1998; Thakur et al. 2021). The function of repetitive DNA, aside from rRNA genes and repeats with structural functions in centromeres and telomeres, remains largely enigmatic. They are considered to have an impact on the maintenance of genomic architecture, proper chromosome segregation and regulation of gene expression (Garrido-Ramos 2015; Mehrotra and Goyal 2014; Šatović-Vukšić and Plohl 2023).

Satellite DNAs, the fast-evolving parts of plant genomes, seem to be an important factor in genome evolution (Biscotti et al. 2015; Schmidt et al. 2024; Shatskikh et al. 2020). Their genomic and chromosomal organisation can vary even among closely related species (He et al. 2024; Jo et al. 2011; Lee et al. 2018; Yücel et al. 2024). Thus, the changes in the genomic composition of satDNAs seem to accompany the diversification and speciation of many taxa and are found to be associated with hybrid incompatibility and meiotic drive (Ferree and Barbash 2009; Fishman and Saunders 2008; Giraud et al. 2021). In various plant groups, the patterns of satDNAs evolution were found to be correlated with taxonomical relationships among species; thus, they can be important tools in phylogenetic and evolutionary studies (Bolsheva et al. 2019; Dodsworth et al. 2014; Ishiguro et al. 2025; McCann et al. 2020; Mehrotra and Goyal 2014; Vitales et al. 2020).

Different models of satellite evolution have been proposed to explain their significant variation in copy number and nucleotide sequence, even among closely related species (Garrido-Ramos 2017; Louzada et al. 2015; Lower et al. 2018). The “library hypothesis” suggests that a group of related species shares a common library of repetitive sequences. However, these repeats are characterised by different amplification levels across species (Fry and Salser 1977; Mestrović et al. 1998; Palacios-Gimenez et al. 2020). Various members of this library can undergo differential amplification or elimination over evolutionary timescales, resulting in species-specific patterns of repetitive DNA distribution (Camacho et al. 2022; Garrido-Ramos 2017; Quesada del Bosque et al. 2011; Samoluk et al. 2017). Tandem repetitive sequences, satDNAs and rRNA genes are considered to undergo concerted evolution, a process in which repetitive DNA sequences evolve in a coordinated manner across multiple copies within a genome, rather than diverging independently due to mutation (Naish and Henderson 2024; Pérez-Gutiérrez et al. 2012; Trunova et al. 2024; Wang et al. 2023). Within arrays, homogenisation occurs through mechanisms such as unequal crossing-over, gene conversion, and replication slippage, maintaining greater similarity among copies within a species than between repeats belonging to the same family in closely related species (Ganley and Kobayashi 2007; Garrido-Ramos 2017; Quesada del Bosque et al. 2011; Šatović-Vukšić and Plohl 2023). Since many satDNAs are species-specific and relatively few satellite families are conserved across genera, another model of sequence evolution, “birth-and-death”, should also be taken into consideration (Nei and Rooney 2005; Subirana et al. 2015). According to the “birth-and-death” model, a new variant of satDNA repeats arises through unequal crossing-over, replication slippage and transposon activity. Then, this new satellite can be highly amplified (“birth”), while other satDNAs can be either eliminated from the genome (e.g., through recombination-based mechanisms) or may accumulate mutations and, as a consequence, lose their repetitive nature (“death “; Belyayev et al. 2020; Lower et al. 2018; Navrátilová et al. 2008; Subirana et al. 2015; Zattera and Bruschi 2022). This hypothesis also predicts the emergence of satellite subfamilies that may contribute to the increase in intragenomic, intraspecific and interspecific diversity of tandem repeats (Belyayev et al. 2020; Subirana et al. 2015; Targueta et al. 2023).

*Crepis sensu lato* (*s.l.*; Asteraceae) comprises approximately 200 annual and perennial species, primarily diploids (Babcock 1947). Two main evolutionary lineages were distinguished: *Lagoseris* and *Crepis sensu stricto* (*s.s.*), with the latter further divided into four main clades of closely related species (Enke et al. 2011; Enke and Gemeinholzer 2008; Senderowicz et al. 2021). *Crepis s.s.* exhibits considerable variation in genome sizes (0.7–7.5 pg/1C DNA among diploids) and base chromosome numbers (*x* = 3–6, 11) (Babcock 1947; Enke et al. 2011; Senderowicz et al. 2021). Additionally, their karyotypes often contain a few relatively large and morphologically well-differentiated chromosomes, making them valuable models for studying chromosome evolution (Babcock 1947; Enke et al. 2015; Senderowicz et al. 2021, 2022; Smocovitis 2009). Recent studies suggested that chromosome number and genome size changes accompanied diversification and speciation in *Crepis* (Enke et al. 2011, 2015; Enke and Gemeinholzer 2008; Senderowicz et al. 2021). Comparative analysis of chromosomal distribution of 5S and 35S rDNA loci revealed multiple chromosome rearrangement events, primarily translocations and inversions, indicating dynamic repatterning of repetitive sequences during *Crepis* evolution (Enke et al. 2015; Senderowicz et al. 2022). This hypothesis was further supported by the analysis of satDNAs in *Lagoseris* lineages. The amplification, elimination, or reorganisation of satDNAs accompanied diversification and speciation in this group of plants. The unique genomic and chromosomal organisation of analysed repeats was revealed for closely related species (Yücel et al. 2024). In *Crepis s.s*. satDNAs have been characterised only in *C. capillaris* (2*n* = 6) using a method based on enzyme restriction and cloning (Jamilena et al. 1993). Three highly abundant satDNAs, pCcE9 (356 bp), pCcD29 (259 bp), and pCcH32 (159 bp), which together constitute approximately 11.5% of the genome, were identified in *C. capillaris* genome (Jamilena et al. 1993). Therefore, the comparative analyses of these three satDNAs in species closely related to *C. capillaris* were analysed in this work to further characterise the repeat evolution in this genus. Specifically, we asked whether these satDNAs are exclusive to *C. capillaris*, supporting the “birth-and-death” hypothesis, or whether they are also present in related species, consistent with the “library hypothesis”. Furthermore, the relatively large chromosomes of *Crepis* provide an opportunity to compare satDNA chromosomal distribution patterns across species. This allows for the assessment of whether each species possesses a unique satDNA genomic and chromosomal organisation or if these sequences are conserved and similarly organised in related species, despite variations in chromosome number inferred from evolutionary analyses (Senderowicz et al. 2021, 2022).

## Materials and methods

### Plant material and DNA isolation

Thirty-five accessions representing 32 *Crepis* species and *Lapsana communis* L. were analysed. Plants were grown from seeds in a greenhouse facility of the University of Silesia in Katowice under a 16 h/8 h photoperiod at 19 ± 2 °C. Vouchers were deposited at the Herbarium KTU (University of Silesia, Chorzów, Poland; Table 1). Total genomic DNA was isolated from fresh leaf tissue using the modified CTAB method (Emadzade et al. 2014). Genomic DNA from each sample was analysed for quality and quantity using a Nanodrop ND-1000 spectrophotometer (peqLab, Erlangen, Germany).

**Table 1:** Species names, collection details, voucher numbers of the analysed taxa, and GenBank accession numbers of the sequences obtained in this study

Species	Collection details	Voucher Number	GenBank accession number		
			pCcE9	pCcD29	pCcH32
***Crepis s.s***					
*C. acuminata* Nutt	USDA-NPGS*; W6 40,086				
*C. albida* Vill	Université Grenoble Alpes, France			PV520844-PV520852	PV520784-PV520788
*C. alpestris* (Jacq.) Tausch	Botanical Garden of Universitat Graz, Austria, N49	KTU157712		PV520843; PV520853-PV520858; PV520905-PV520908	PV520767-PV520771
*C. alpina* L	USDA-NPGS^*^; PI 274367	KTU154609			
*C. atribarba* A.Heller	USDA-NPGS^*^; W6 36,843				
*C. aurea* (L.) Cass	USDA-NPGS^*^; PI 312843	KTU157719		PV520886-PV520899	PV520780-PV520783; PV520795
*C. biennis* L	Berlin-Dahlem Botanical Garden, Germany; 656	KTU154629			
*C. capillaris* Wallr	The Botanical Garden of Göttingen University, Germany, 335	KTU154610	PV520719-PV520723	PV520796-PV520811	PV520747-PV520756
*C. conyzifolia* (Gouan) A.Kern	Giardino Botanico Alpino "Rezia", Italy, 462	KTU157720			
*C. conyzifolia subsp. djimilensis* (K.Koch) Lamond	Batumi Botanical Garden, Georgia				
*C. foetida subsp. rhoaedifolia* (M.Bieb.) Celak	Hortus Botanicus Budapest, Hungary 1734	KTU154614			
*C. kotschyana* Boiss	USDA-NPGS^*^; PI 310392	KTU164608			
*C. modocensis* Greene	USDA-NPGS^*^; W6 49,189				
*C. mollis* Asch	Sławków, Poland N 50°17′45.90″ E 19°16′59.06″	KTU154630			
*C. leontodontoides* All	Berlin-Dahlem Botanical Garden, Germany; 658	KTU154631		PV520833-PV520842	
*C. oporinoides* Boiss. ex Froel	Alpine Botanical Garden of Lautaret, France; 1516	KTU154622			
*C. paludosa* Moench	Sławków, Poland N 50°18′07.51″ E 19°21′19.10″	KTU154625			
*C. pannonica* (Jacq.) K.Koch 1	Botanical Garden Freie Universität Berlin—Dahlem; Germany, 256–01-00–14	KTU154627			
*C. polymorpha* Pourr	Jardin botanique de Nancy; France,149	KTU157725	PV520729-PV520733	PV520831-PV520832; PV520875; PV520879-PV520884; PV520909	PV520762-PV520763
*C. pygmaea* L	Université Grenoble Alpes; France, 239	KTU157722			
*C. pyrenaica* (L.) Greuter	Berlin-Dahlem Botanical Garden, Germany; 1010	KTU154621		PV520900-PV520904; PV520910-PV520915	PV520789-PV520794
*C. setosa* Haller f. 1	Hortus Botanicus Universitatis, Romania, 1275	KTU154620			
*C. sibirica* L	Berlin-Dahlem Botanical Garden, Germany; 738	KTU157721			
*C. succisifolia* Tausch	Rędziny, Poland N 50°49′08.66″ E 15°55′55.27″	KTU154656			
*C. syriaca* (Bornm.) Babc. & Navashin	Millenium Seed Bank KEW Gardens; 0129064	KTU154615			
*C. taraxacifolia* Thuill	Botanical Garden of Göttingen University; 347	KTU157723	PV520723-PV520728	PV520812-PV520820; PV520885	PV520757-PV520761; PV520779
*C. tectorum* L	Ustroń, Poland N 49°43′14.68″ E 18°49′29.11″	KTU157717			
*C. vesicaria* L. 3	Orto Botanico dell Universito di Padora, Italy	KTU157724	PV520734-PV520738	PV520830; PV520859-PV520863; PV520876-PV520879	PV520764; PV520772-PV520776
*C. vesicaria* L. 2	Berlin-Dahlem Botanical Garden; Germany; 918	KTU157726		PV520826-PV520829; PV520864; PV520866-PV520870	
*C. veiscaria* L. 1	Berlin-Dahlem Botanical Garden; Germany; 1014	KTU154616	PV520739-PV520746	PV520821-PV520825; PV520871-PV520875	PV520765-PV520766; PV520777-PV520778
*C. zacintha* (L.) Loisel	Botanic Garden of Tel Aviv University, Israel; 92	KTU154606			
***Lagoseris***					
*C. palaestina* Bornm	The Botanical Garden of Göttingen University, Germany; 335	KTU154611			
*C. pulchra* L	The Botanical Garden of Göttingen University, Germany; 341	KTU154648			
*C. preamorsa* (L.) Tausch	Berlin-Dahlem Botanical Garden, Germany; 662	KTU154628			
*C. sancta* (L.) Bornm	Botanischer Garten Universität Konstanz, Germany; 104	KTU154613			
*Lapsana communis* L. 1	Millennium Seed Bank, KEW Gardens; Great Britain; 0018568	KTU154617			

^*^USDA-NPGS—USDA North central regional plant introduction station of the US national plant germplasm system

### DNA amplification, cloning and sequencing

Three tandem repetitive sequences (pCcE9, pCcD29 and pCcH32; Jamilena et al. 1993) were amplified according to Yücel et al. (2024). Primer sequences and MgCl_2_ concentrations were listed in Supplementary Table 1. The PCR amplification protocol consisted of an initial denaturation step of 3 min at 94 °C, followed by 35 cycles of amplification consisting of 1 min denaturation at 94 °C, 50 s for primer annealing at 52/55 °C (Supplementary Table 1), and 50 s of DNA extension at 72 °C. The amplicons were cloned using the pGEM-T Easy vector system (Promega, Madison, USA) following the manufacturer’s instructions. The individual clones were sequenced by Macrogen (Amsterdam, The Netherlands). At least five clones were analysed for each accession from which the given satDNA family could be amplified. All sequences were deposited in GenBank (Table 1).

### Dot blot hybridisation and Southern hybridisation

The analyses were carried out using DIG-High Prime DNA Labelling and Detection Starter Kit I (SIGMA, St. Louis, Missouri, USA). In dot blot hybridisation, the genomic DNA of each species (0.5 µg) was denatured at 95 °C and blotted onto a positively charged nylon membrane (Roche, Mannheim, Germany) using the Dot Blot 96 System (Biometra, Gottingen, Germany). DNA was UV cross-linked to the membrane using a CK-1000 Ultraviolet Crosslinker (Ultra-Violet Products, Cambridge, UK). Satellite repeats were labelled by nick translation with alkali labile digoxigenin-11-dUTP (Roche, Basel, Switzerland) according to the manufacturer’s instructions. The hybridisation was conducted overnight at 37 °C. Then, the membrane was washed in 1 × SSC (saline sodium citrate buffer) with 0.1% SDS at 65 °C (stringency: 83% for pCcE9, 81% for pCcD29, 83% for pCcH32). Hybridisation signals were documented using ChemiDocXRS (BioRad, Hercules, CA, USA).

For Southern hybridisation, approximately 2–4 µg of DNA of the analysed species was digested with restriction enzymes *Taq*I or *Alu*I. Fragments were separated by electrophoresis in 1% w/v agarose gel and then transferred to a positively charged nylon membrane (Roche, Basel, Switzerland) using a Vacu-blot system (Biometra, Gottingen, Germany). The probe labelling, hybridisation, and signal detection were performed as described for dot blot hybridisation. Post-hybridisation washes were performed in 3.5 × or 1 × SSC with 0.1% SDS at 65 °C (stringency: 74% for pCcE9, 72% and 83% for pCcH32; 81% for pCcD29).

### Chromosome preparation and fluorescent *in situ* hybridisation

Young leaves from the analysed species were used as a material for cytogenetic slide preparation. Leaves were pretreated with 2 mM 8-hydroxyquinoline for 2 h at room temperature and 2 h at 4 °C, fixed in methanol: glacial acetic acid (3:1) and stored at −20 °C until used. Mitotic metaphase chromosome spreads were prepared as described earlier (Senderowicz et al. 2021).

Formamide-free fluorescent in situ hybridisation (FISH) was performed according to Jang and Weiss-Schneeweiss (2015) with modifications. The satDNAs (Jamilena et al. 1993) were used as DNA probes and labelled by nick translation with digoxigenin-11-dUTP (Roche, Basel, Switzerland). Additionally, the 25S rDNA coding region of *Arabidopsis thaliana* (Unfried and Gruendler 1990) labelled with tetramethyl-rhodamine-5-dUTP (Roche, Basel, Switzerland), was used as a chromosomal marker to facilitate the identification of homologous chromosome pairs. Hybridisation mixture, consisting of 100 ng of each labelled DNA probe, 10% dextran sulphate, 0.02 × SSC, 1% sonicated salmon sperm DNA, was denatured for 10 min at 95 °C and immediately cooled down on ice. Chromosome spreads were denatured on an Omnislide Thermal cycler (ThermoHybaid, Franklin, MA, USA) at 72 °C for 4 min with 70% formamide in 2 × SSC. The slides were immediately transferred to ice-cold 75% and 100% ethanol and air-dried. Hybridisation was carried out for 48 h at 37 °C in a humid chamber. Post-hybridisation washes (2 × SSC at 37 °C) were followed by the detection of digoxigenin with FITC-conjugated primary anti-digoxigenin sheep antibody (Roche, Basel, Switzerland). Signal amplification was performed with the use of a secondary antibody (FITC-conjugated anti-sheep antibody; Jackson ImmunoResearch, West Grove, PA, USA). The chromosome slides were stained with 2 μg/ml DAPI (4′,6-diamidino-2-phenylindole) in Vectashield (Vector Laboratories, Peterborough, UK) and analysed under an epifluorescent microscope Zeiss AxioImager.Z.2 (ZEISS, Aalen, Germany) equipped with an AxioCam HRm monochromatic camera (Zeiss, Germany).

### Sequence alignment and phylogenetic analysis

The phylogenetic analysis was performed using the cloned DNA sequences of satDNAs obtained in this study. Multiple sequence alignment was performed 20 times using webPRANK (Löytynoja and Goldman 2010), and MergeAlign (Collingridge and Kelly 2012) was used to obtain a consensus multiple sequence alignment. Phylogenetic relationships were conducted using maximum likelihood (ML) analyses as implemented in IQ-TREE version 2.2.2.6 (Minh et al. 2020). Best-fitted models of sequence evolution among analysed species were TPM2 + F for pCcE9, K3Pu + F + I + G4 for pCcD29 and K3Pu + F + G4 for pCcH32 according to IQ-TREE. The significance of the inferred relationships was assessed via bootstrapping with 1000 replicates.

## Results

### Genomic organisation and chromosomal localisation of pCcD29 in *Crepis*

**Fig. 1:**
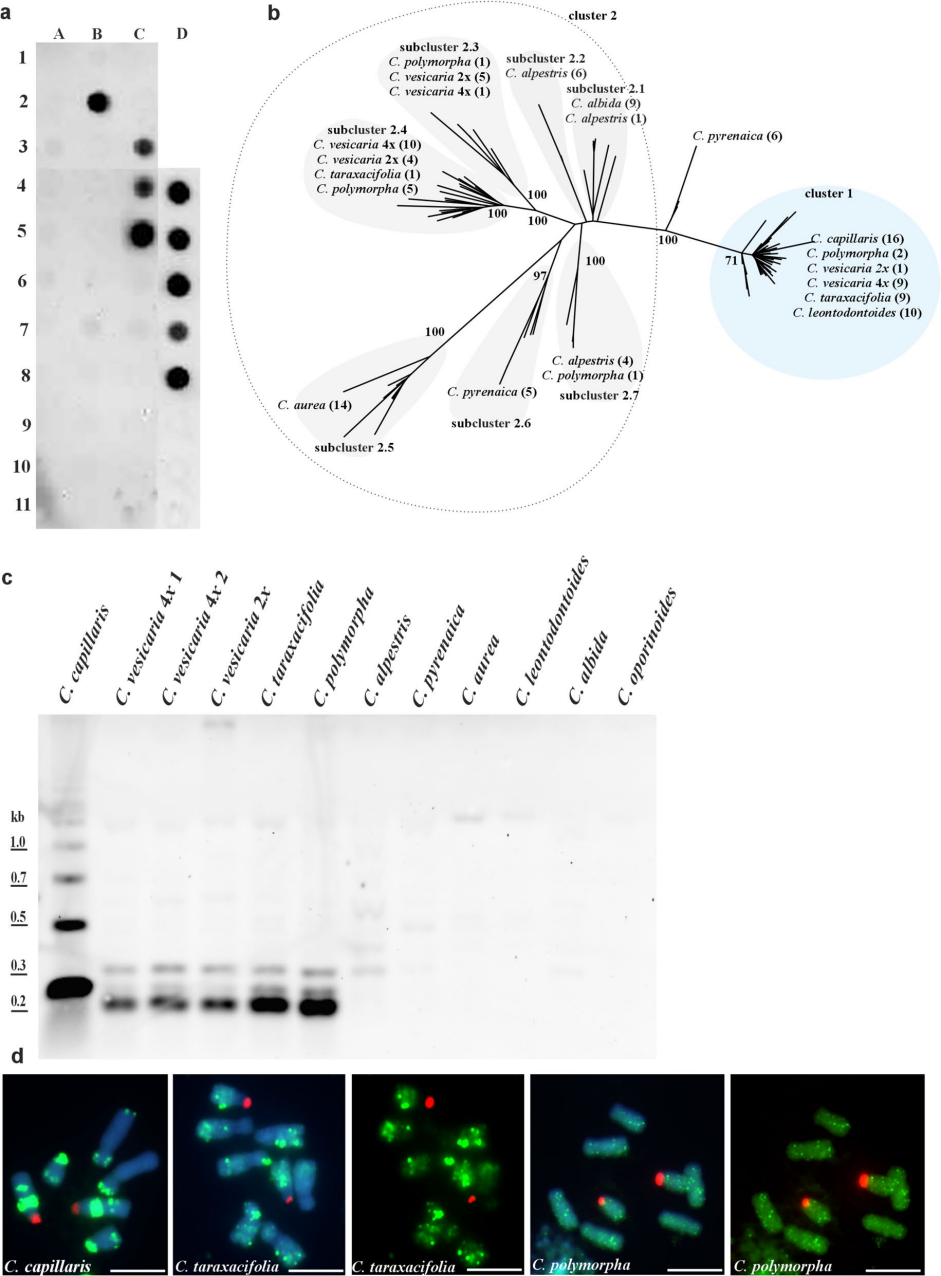
Organisation of pCcD29 satDNA family in genomes of analysed species. (**a**) Dot-blot hybridisation revealing the presence of pCcD29 sequence isolated from *C. capillaris* in different *Crepis* species: *C. zacintha* (A1), *C. tectorum* (A2), *C. biennis* (A3), *Lapsana communis* (A4), *C. sancta* (A5), *C. palaestina* (A6), *C. pulchra* (A7), *C. paludosa* (A8), *C. mollis* (A9), *C. succisifolia* (A10), *C. pygmaea* (A11), *C. kotschyana* (B1), *C. capillaris* (B2), empty slot (B3), *C. atribarba* (B4), *C. acuminata* (B5), *C. modocensis* (B6), empty slot (B7), *C. praemorsa* (B8), empty slot (B9), *C. sibirica* (B10), empty slot (B11), *C. alpestris* (C1), *C. setosa* (C2), *C. taraxacifolia* (C3), *C. vesicaria* 1 (C4), *C. polymorpha* (C5), *C. pyrenaica* (C6), *C. oporinoides* (C7), *C. conyzifolia* subsp*. dijmilensis* (C8), *C. conyzifolia* (C9), *C. pannonica* (C10), *C. albida* (C11), empty slot (D1-D3), *C. vesicaria* 1 (D4), *C. vesicaria* 2 (D5), *C. vesicaria* 3 (D6), *C. taraxacifolia* (D7), *C. polymorpha* (D8), empty slot (D9-D11); (**b**) The unrooted phylogenetic tree of the cloned pCcD29 repeats isolated from the studied *Crepis* species inferred through maximum likelihood analysis. Numbers in parentheses indicate the number of analysed clones. Bootstrap support scores ≥ 70% are shown near branches; (**c**) Southern hybridisation of pCcD29 to *Taq*I—restricted genomic DNA of analysed species (stringency 81%); (**d**) Metaphase plates of analysed species representing chromosomal distribution of pCcD29 sequence. Fluorescent in situ hybridisation with pCcD29 (green fluorescence) and 25S rDNA (red fluorescence) as DNA probes to chromosomes of selected *Crepis* species, scale bar = 5 µm

Distribution of pCcD29 repeat-like sequences was analysed in genomes of 30 species representing four clades of *Crepis s.s.* and *Lagoseris* lineage (Supplementary Fig. 1). Dot blot hybridisation (stringency 81%) with a 262 bp long clone isolated from *C. capillaris* as a probe, revealed intense signals for *C. capillaris* and three other closely related species (*C. taraxacifolia, C. polymorpha* and both diploid and tetraploid *C. vesicaria* accessions; Fig. 1a), showing that these satellite families were amplified also in genomes of related species. Since the dot blot revealed only the presence of highly amplified sequences, a PCR was used to amplify pCcD29-like sequences from other *Crepis* species. Attempts to isolate these sequences were successful for the following species: *C. alpestris*, *C. pyrenaica*, *C. aurea*, *C. leontodontoides*, and *C. albida*, all closely related to *C. capillaris*. The resulting alignment was 482 bp long (including gaps), with 240 characters identified as parsimony-informative. The length of analysed sequences in the alignment ranged from 156 to 366 bp due to large InDels up to 202 bp in *C. aurea*.

Maximum likelihood analysis revealed a deep split between two major clusters of these repeats (BS = 100; Fig. 1b), with several sequences isolated from *C. pyrenaica* (approximately 320 bp long) positioned along the split. The first cluster consisted of sequences whose length ranged from 253 to 279 bp isolated from *C. taraxacifolia*, *C. capillaris, C. leontodontoides*, *C. vesicaria* (both diploid and tetraploid) and *C. polymorpha* (BS = 71). The second cluster (BS = 100) consisted of sequences isolated from all analysed species except *C. capillaris* and *C. leontodontoides* with lengths ranging from 156 to 366 bp. Most sequences included in cluster 1 showed a high similarity (above 90%), while sequences from cluster 2 showed higher diversity. The second cluster could be further divided into seven subclusters: (i) sequences from *C. alpestris* and *C. albida* (approximately 338 bp, subcluster 2.1, similarity above 84%); (ii) sequences from *C. alpestris* (BS = 100; approximately 342 bp, subcluster 2.2, similarity above 91%); (iii) sequences from *C. polymorpha* and diploid and tetraploid accession of *C. vesicaria* (BS = 100; approximately 345 bp, subcluster 2.3, similarity above 84%); (iv) sequences from *C. taraxacifolia, C. polymorpha* and diploid and tetraploid accession of *C. vesicaria* (BS = 100; approximately 303 bp, subcluster 2.4, similarity above 62%); (v) sequences from *C. aurea* (BS = 100, approximately 158 bp, subcluster 2.5, similarity above 80%); (vi) sequences from *C. pyrenaica* (BS = 97, approximately 347 bp, subcluster 2.6, similarity above 79%); (vii) sequences from *C. alpestris* and *C. polymorpha* (BS = 100; approximately 342 bp, subcluster 2.7, similarity above 83%).

Nine different variants of the pCcD29-like sequence were identified in the analysed species. More than one variant of the satDNA was present in genomes of five species (*C. pyrenaica, C. alpestris, C. taraxacifolia, C. polymorpha* and both diploid and tetraploid accessions of *C. vesicaria*), while for four other species, *C. capillaris, C. leontodontoides, C. albida* and *C. aurea*, only one variant was detected.

After Southern hybridisation (stringency 81%) of pCcD29 probe to *Taq*I-digested genomic DNA, the intense hybridisation signals were obtained for *C. capillaris*, *C. taraxacifolia*, *C. polymorpha* and diploid and tetraploid accessions of *C. vesicaria* (Fig. 1c). Very weak hybridisation signals were also visible for *C. alpestris*, *C. pyrenaica*, *C. albida, C. leontodontoides* and *C. aurea,* indicating a low amplification level of this repeat (Fig. 1c). Southern hybridisation showed that this sequence has different genomic organisation in the analysed species. A ladder-like pattern, with the shortest bands corresponding to the size of the monomer (~ 262 bp), typical for satellite DNA, was observed in *C. capillaris* (Fig. 1c). A different pattern of signals was observed for three closely related species: *C. taraxacifolia, C. polymorpha*, and *C. vesicaria* (both diploid and tetraploid accessions). This pattern, consisting of three bands (approximately 210 bp, 250 bp, and 340 bp), revealed that different length variants of the pCcD29 satellite DNA family exist in their genomes, which is congruent with cloning experiments. The *Taq*1 enzyme cuts the pCcD29 sequence present in *C. capillaris* once. However, the sequence analysis of the repeat variants (present in *C. taraxacifolia, C. polymorpha*, and *C. vesicaria*) revealed the presence of an additional restriction site (Supplementary Fig. 2a). Thus, the bands corresponding to 213 bp and 132 bp long sequences can be expected. Indeed, a fast-migrating band of approximately 200 bp was present in the *C. vesicaria* group. This band represented sequences from subcluster 2.4. The bands of approximately 250 bp represented sequences from cluster 1 (Fig. 1b), while the longest band (~ 340 bp) corresponded to the sequences from subcluster 2.3. The intensity of the observed bands was not uniform. The most striking differences were observed for the variant of 250 bp, which was highly amplified in *C. capillaris*, whereas in the other species, less intense bands of this length were observed.

FISH using 25S rDNA and pCcD29 as probes was performed on *C. capillaris, C. taraxacifolia, C. polymorpha*, and both diploid and tetraploid accessions of *C. vesicaria*. In *C. capillaris*, five to six distinct loci of pCcD29 were observed (Fig. 1d). Different patterns of hybridisation signals were observed in C. *polymorpha* and *C. taraxacifolia*. In *C. polymorpha*, numerous small signals were dispersed along the chromosome arms (Fig. 1d). *C. taraxacifolia* exhibited numerous minor and several major loci of pCcD29 distributed across all chromosomes; however, most of the signals were placed in interstitial and/or subterminal parts of long chromosome arms (Fig. 1d). No hybridisation signals were observed in all *C. vesicaria* accessions.

### Genomic organisation and chromosomal localisation of pCcE9 in Crepis

**Fig. 2:**
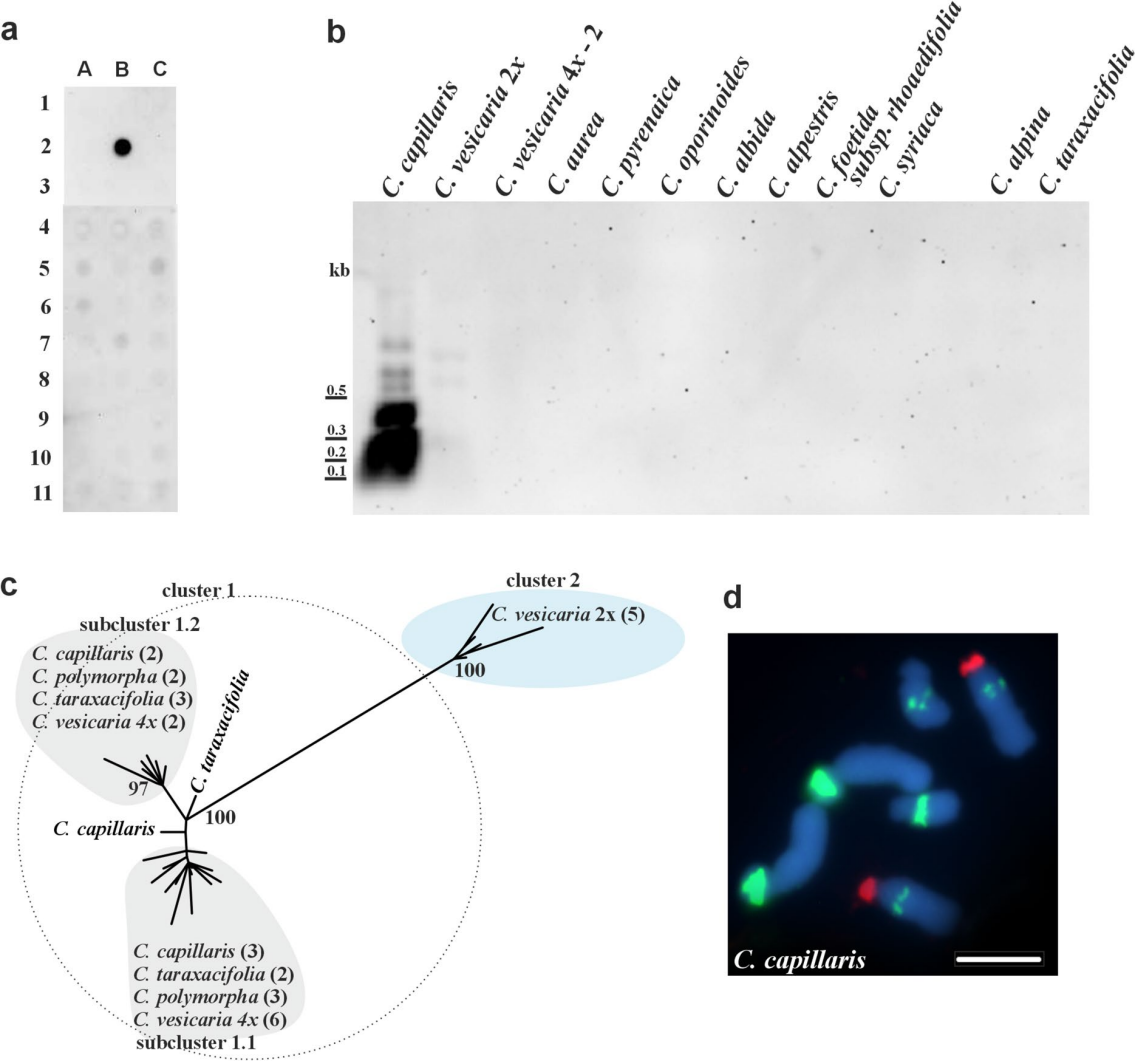
Organisation of pCcE9 satDNA family in genomes of analysed species. (**a**) Dot-blot hybridisation revealing the presence of pCcE9 sequence isolated from *C. capillaris* in different *Crepis* species: *C. zacintha* (A1), *C. tectorum* (A2), *C. biennis* (A3), *Lapsana communis* (A4), *C. sancta* (A5), *C. palaestina* (A6), *C. pulchra* (A7), *C. paludosa* (A8), *C. mollis* (A9), *C. succisifolia* (A10), *C. pygmaea* (A11), *C. kotschyana* (B1), *C. capillaris* (B2), empty slot (B3), *C. atribarba* (B4), *C. acuminata* (B5), *C. modocensis* (B6), empty slot (B7), *C. praemorsa* (B8), empty slot (B9), *C. sibirica* (B10), empty slot (B11), *C. alpestris* (C1), *C. setosa* (C2), *C. taraxacifolia* (C3), *C. vesicaria* 1 (C4), *C. polymorpha* (C5), *C. pyrenaica* (C6), *C. oporinoides* (C7), *C. conyzifolia* subsp*. djimilensis* (C8), *C. conyzifolia* (C9), *C. pannonica* (C10), *C. albida* (C11); (**b**) Southern hybridisation of pCcE9 satDNA to *Alu*I—restricted genomic DNA of analysed species (stringency 74%); (**c**) The unrooted phylogenetic tree of the cloned pCcE9 repeats isolated from the studied *Crepis* species inferred through maximum likelihood analysis. Numbers in parentheses indicate the number of analysed clones. Bootstrap support scores ≥ 70% are shown near branches; (**d**) FISH with pCcE9 repeat (green fluorescence) and 25S rDNA (red fluorescence) to chromosomes of *C. capillaris,* scale bar = 5 µm

Distribution of pCcE9 repeat (356 bp long) was analysed in genomes of 30 species representing four clades of *Crepis s.s.* and *Lagoseris* lineage (Supplementary Fig. 1). Dot blot hybridisation (stringency 83%) revealed an intense pCcE9 signal only for *C. capillaris* (Fig. 2a). After Southern hybridisation (stringency 74%) of the pCcE9 probe to *Alu*I-digested genomic DNA, the intense hybridisation signals were obtained for *C. capillaris*. Very weak hybridisation signals were also visible for diploid *C. vesicaria*, indicating a low amplification level of this repeat, while no hybridisation signals were observed for other selected species (Fig. 2b). Southern hybridisation was not performed for *C. polymorpha* due to a lack of material. The *Alu*I restriction enzyme cut the monomer sequence twice (positions: 94 bp and 311 bp; Supplementary Fig. 2b). Strong bands of approximately 120 bp and 220 bp (representing fragments cut twice with restriction enzymes), as well as a band 340 bp long (unit cut once with *Alu*I), were observed after hybridisation (Fig. 2b and Supplementary Fig. 2). This pattern supported the tandem repeat nature of this sequence family. The presence of sequences related to pCcE9 was tested in the selected *Crepis* species, closely related to *C. capillaris,* with PCR amplification and cloning. Sequences homologous to pCcE9 were amplified only from genomes of three species: *C. taraxacifolia, C. polymorpha,* diploid and tetraploid accessions of *C. vesicaria*. The alignment of pCcE9 family sequences was 394 bp long (including gaps) with 81 characters that were parsimony informative. The length of analysed sequences in all studied species ranged from 303 to 364 bp. ML analysis revealed a deep split between two major clusters of repeats (Fig. 2c).

The first distinguished cluster consisted of sequences whose lengths range from 303 to 364 bp isolated from diploid accessions of *C. vesicaria* (BS = 100). The second cluster (BS = 100) consisted of sequences with lengths ranging from 334 to 356 bp isolated from all other studied species. Each of these two groups consisted of sequences with an identity higher than 88%. The similarity between these two sequence clusters ranged from 71 to 81%. In the second cluster, two subclusters were distinguished, each consisting of sequences from *C. capillaris, C. taraxacifolia, C. polymorpha* and a polyploid accession of *C. vesicaria.*

FISH using 25S rDNA and pCcE9 as probes was performed on *C. capillaris*. Three major loci of pCcE9 were observed. The strongest hybridisation signals were detected on the distal/subtelomeric region of the short arms of the longest chromosome pair. In the other two chromosome pairs, the hybridisation signals were detected in the interstitial position in the long chromosome arms (Fig. 2d).

### Genomic organisation and chromosomal localisation of pCcH32 in *Crepis*

**Fig. 3:**
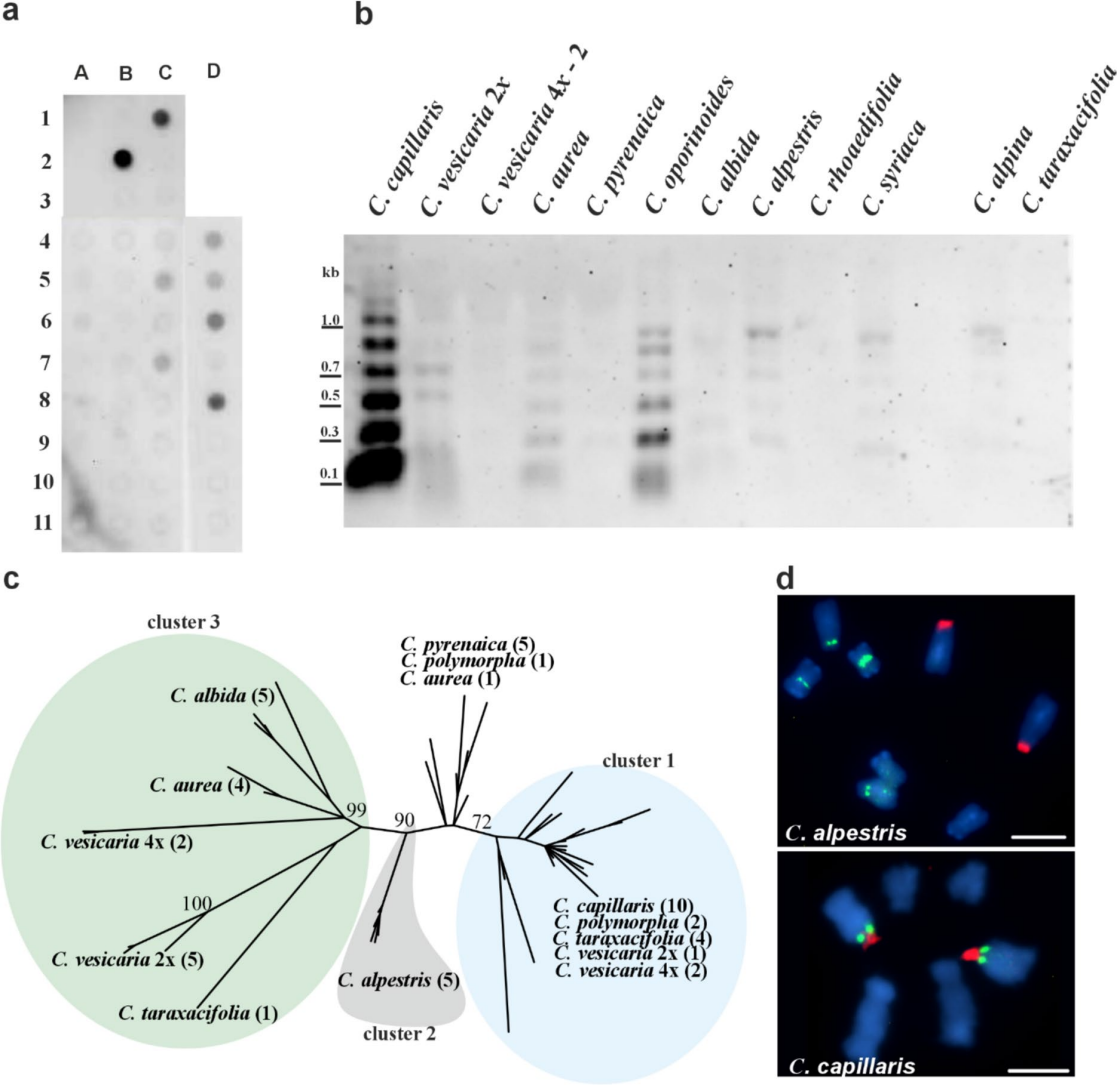
Organisation of pCcH32 satDNA family in genomes of analysed species. (**a**) Dot-blot hybridisation revealing the presence of pCcH32 sequence isolated from *C. capillaris* in different *Crepis* species: *C. zacintha* (A1), *C. tectorum* (A2), *C. biennis* (A3), *Lapsana communis* (A4), *C. sancta* (A5), *C. palaestina* (A6), *C. pulchra* (A7), *C. paludosa* (A8), *C. mollis* (A9), *C. succisifolia* (A10), *C. pygmaea* (A11), *C. kotschyana* (B1), *C. capillaris* (B2), empty slot (B3), *C. atribarba* (B4), *C. acuminata* (B5), *C. modocensis* (B6), empty slot (B7), *C. praemorsa* (B8), empty slot (B9), *C. sibirica* (B10), empty slot (B11), *C. alpestris* (C1), *C. setosa* (C2), *C. taraxacifolia* (C3), *C. vesicaria* 1 (C4), *C. polymorpha* (C5), *C. pyrenaica* (C6), *C. oporinoides* (C7), *C. conyzifolia* subsp*. djimilensis* (C8), *C. conyzifolia* (C9), *C. pannonica* (C10), *C. albida* (C11), empty slot (D1-D3), *C. vesicaria* 1 (D4), *C. vesicaria* 2 (D5), *C. vesicaria* 3 (D6), *C. taraxacifolia* (D7), *C. polymorpha* (D8), empty slot (D9-D11); (**b**) Southern hybridisation of isolated pCcH32 repeat to *Alu*I – restricted genomic DNA of analysed species (stringency 72%); (**c**) The unrooted phylogenetic tree of the cloned pCcH32 repeats isolated from the studied *Crepis* species inferred through maximum likelihood analysis. Bootstrap support scores ≥ 70% are shown near branches; Numbers in parentheses indicate the number of analysed clones; (**d**) FISH with pCcH32 repeat (green fluorescence) and 25S rDNA (red fluorescence) to chromosomes of *C. capillaris* and *C. alpestris,* scale bar = 5 µm

Distribution of pCcH32 repeat (159 bp long) was analysed in genomes of 30 species representing four clades of *Crepis s.s.* and *Lagoseris* lineage (Supplementary Fig. 1). Dot blot hybridisation revealed an intense signal for pCcH32 in the genome of *C. capillaris* and *C. alpestris* (Fig. 3a). Several species (*C. polymorpha*, *C. oporinoides*, diploid and polyploid accessions of *C. vesicaria*) showed weaker signals, suggesting a lower level of sequence amplification in their genomes. The genomic organisation of pCcH32 was analysed in genomes of selected species, closely related to *C. capillaris*, using Southern hybridisation. Hybridisation of the pCcH32 probe (stringency 83%; Supplementary Fig. 3) to *Alu*I-digested genomic DNA revealed strong hybridisation signals only for *C. capillaris* (Supplementary Fig. 3) and much weaker ones in diploid *C. vesicaria*, while a hybridisation experiment with lower stringency (72%; Fig. 3b) showed that repeats similar to pCcH32 were amplified also in other species. A relatively strong hybridisation signals were observed for *C. oporinoides,* while much weaker signals were revealed for a diploid accession of *C. vesicaria, C. aurea, C. alpestris, C. syriaca, C. albida* and *C. alpina*, suggesting a low amplification level of pCcH32-like sequences in these species. A ladder-like pattern, with the shortest bands corresponding to the size of the monomer (~ 159 bp), typical for satellite DNA, was observed in all these species (Fig. 3b, Supplementary Fig. 2c). Southern hybridisation was not performed for *C. polymorpha* due to a lack of material.

Sequences similar to pCcH32 were amplified from genomes of eight species closely related to *C. capillaris* (diploid and tetraploid accessions of *C. vesicaria*, *C. taraxacifolia*, *C. polymorpha, C. alpestris, C. pyrenaica, C. albida* and *C. aurea*). The obtained alignment was 187 bp long (including gaps) with 94 characters that were parsimony informative. The length of analysed sequences in all studied species ranged from 136 to 176 bp. ML analysis revealed three major groups of repeats (Fig. 3c). The first group consisted of sequences whose length ranged from 136 to 159 bp isolated from *C. capillaris*, *C. polymorpha*, C*. taraxacifolia* and diploid and polyploid accessions of *C. vesicaria* (BS = 72). The second group (BS = 90) consisted of sequences isolated from *C. alpestris* (BS = 90, approximately 159 bp long). The third group consists of sequences from *C. aurea, C. albida, C. taraxacifolia* and diploid and polyploid accessions of *C. vesicaria* (BS = 99, approximately 162 bp).

FISH using 25S rDNA and pCcH32 as probes was performed on *C. capillaris* and *C. alpestris*. One major locus of pCcH32 was observed in *C. capillaris* chromosomes, located in the pericentromeric region of the NOR chromosome (Fig. 3d). Hybridisation signals for pCcH32 observed in *C. alpestris* showed different chromosome distribution. One signal was observed in the pericentromeric region of the short arm of the subtelocentric chromosome, and the second one was observed in the primary constriction of the submetacentric chromosome (Fig. 3d). No hybridisation signals were observed in diploid *C. vesicaria* and *C. aurea*. FISH was not performed on *C. oporinoides* chromosomes due to the lack of material.

## Discussion

**Fig. 4:**
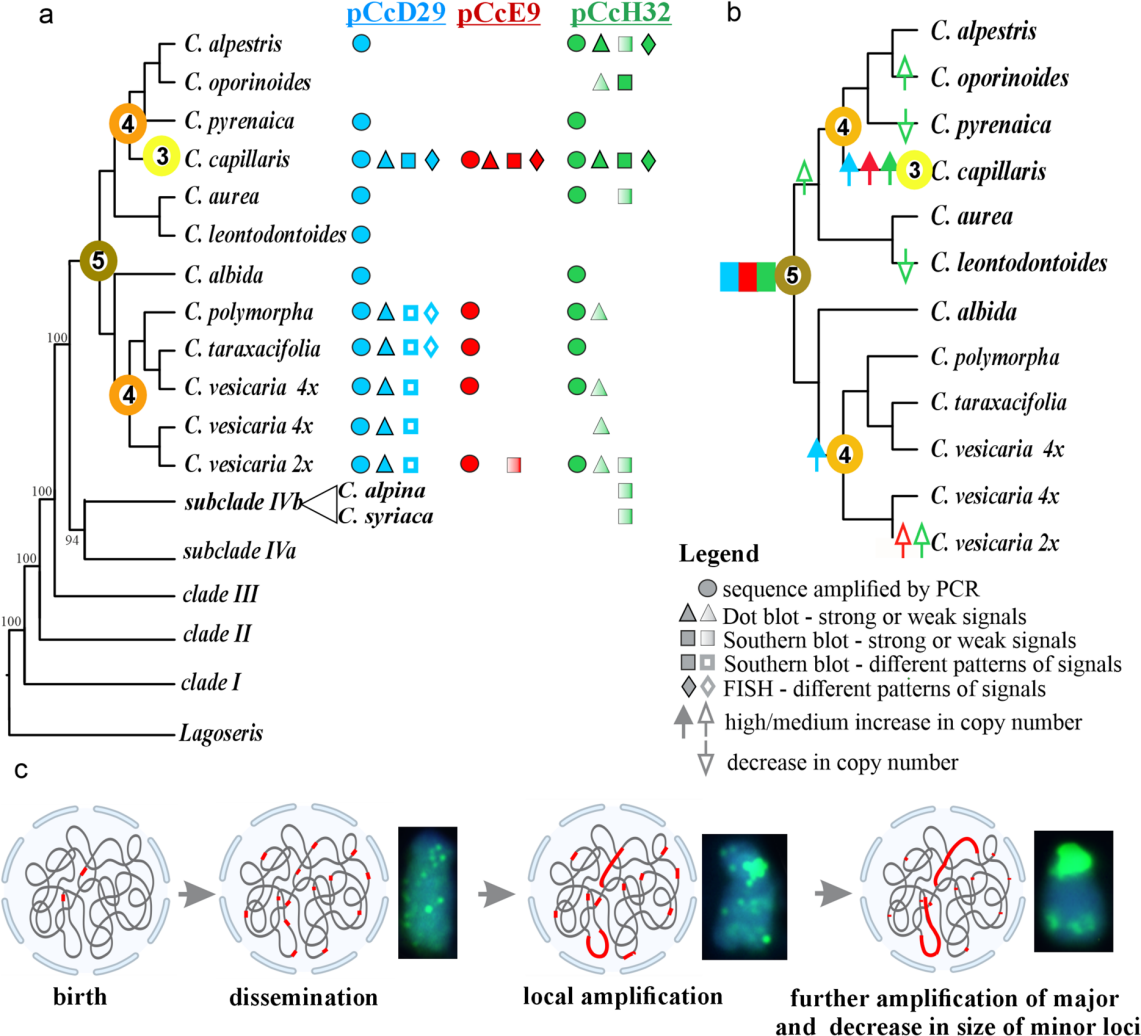
Organisation and evolution of satDNA families in analysed *Crepis s.s.* species. Phylogenetic relationships among the analysed *Crepis* species were obtained based on the cpDNA data sets (Senderowicz et al. 2021). The numbers in circles that represent the ancestral base chromosome number were taken from Senderowicz et al. (2021). BS values are indicated at each node. The tree was rooted with *Picris hieracioides*, *Lactuca serriola*, and *Sonchus oleraceus*, but this outgroup was deleted from the figure. (**a**) Distribution and organisation of satDNA families in *Crepis* species within a phylogenetic context. The satDNA families pCcD29, pCcE9, and pCcH32 are represented in blue, red, and green, respectively. The presence of each repeat family is indicated by different symbols: a circle denotes confirmation by PCR amplification, a triangle indicates detection by dot blot, a square represents evidence from Southern blot, and a diamond shows patterns of chromosomal distribution as revealed by FISH; (**b**) The hypothetical evolutionary trajectories of analysed satDNAs in *Crepis s.s.* The upward arrows indicate the increase in copy numbers, while the downward arrows indicate a decrease in copy numbers (solid arrowhead denotes a major change in copy number, while a hollow arrowhead depicts a minor change). (**c**) satDNA evolution in *Crepis* chromosomes according to the hypothesis of Ruiz-Ruano et al. (2016): The emergence of a satDNA family begins with de novo duplication, followed by the dissemination of short repeat arrays across the genome (e.g., the chromosomal distribution of pCcD29 in *C. polymorpha*). Subsequent local amplification of satDNA results in the formation of major satDNA loci (e.g., the chromosomal distribution of pCcD29 in *C. taraxacifolia*). The repeats in these major loci undergo further amplification and concerted evolution, leading to high sequence homogeneity. In contrast, the number of repeats in minor loci gradually decreases, often falling below the detection threshold of FISH; Created with BioRender.com

Satellite DNAs can arise from any DNA sequence in the genome, and such repeats typically exhibit high rates of evolution both in monomer sequence and overall abundance (Garrido-Ramos 2017; Liu et al. 2024; Macas et al. 2010). Their amplification is often species-specific or limited to closely related species, though some satDNA families are common to entire genera or can even be shared among multiple genera (Félix et al. 2024; Garrido-Ramos 2017; McCann et al. 2020; Orzechowska et al. 2018; Rosato et al. 2011). According to the "library hypothesis", related species share an ancestral set of conserved satellite DNA families that may be differentially amplified in each lineage due to stochastic processes associated with concerted evolution (Mestrović et al. 1998; Salser et al. 1976). Consistent with this hypothesis, sequences similar to the analysed satDNA families were identified in several related species, and in the case of pCcH32, even in species belonging to a different subclade (Fig. 4a).

It should be noted that the absence of PCR amplification for certain satDNA families may result from rapid sequence divergence, which can hinder primer annealing. The high amplification of the analysed satDNA families, however, was restricted only to one (in the case of pCcE9) or very few species (e.g., pCcD29 and pCcH32). The latter satDNA families differed in their species-specific amplification patterns. Sequences similar to pCcH32 were amplified not only in some species closely related to *C. capillaris* but also in genomes of two species from a different subclade (Fig. 4a). Sequences similar to pCcD29 were amplified within the *C. vesicaria* group, but were low abundant/absent in the genomes of *C. capillaris* closest relatives. These results showed that the amplification patterns of both satDNA families do not follow phylogenetic relationships, suggesting independent amplification events and lineage-specific dynamics. Similar patterns of satDNA amplification have previously been reported in species from *Lagoseris* lineage of *Crepis s.l.* (Yücel et al. 2024) and in species from the tribe Cardueae (Asteraceae) (Quesada del Bosque et al. 2013). However, in many other plant groups, the amplification patterns of satDNAs reflect evolutionary history, making repeatome data a useful tool in molecular phylogenetic analyses (Dodsworth et al. 2014; McCann et al. 2020; Vitales et al. 2020).

Phylogenetic analyses revealed that members of the satDNA families in *Crepis* are highly polymorphic and each of them can be split into two or more subfamilies. A similar phenomenon was reported for *Hinf*I satellite repeats in the genomes of major evolutionary lineages within the Cardueae (Asteraceae; del Bosque et al. 2014; Quesada del Bosque et al. 2013). In *Crepis* different subfamilies were differentially amplified depending on the species, as shown for the pCcD29 family. In species of the *C. vesicaria* group, distinct variants were more highly amplified than in *C. capillaris*. However, the species from the *C. vesicaria* group also exhibited amplification, albeit to a lesser extent, of the subfamily predominantly amplified in *C. capillaris*. Southern blot showed similar patterns of pCcH32 hybridisation signals in several analysed species, but the signals were only detected under low-stringency conditions in species other than *C. capillaris,* indicating diversification within this satDNA family (Fig. 4a). In the case of the pCcH32 family, different variants with sequence similarity lower than 82%, compared to the repeats isolated from *C. capillaris,* were amplified in other species. These results demonstrated the diversification of satellite DNA into multiple subfamilies, followed by lineage-specific amplification patterns.

Satellite repeats can exhibit diverse chromosomal distributions depending on the satDNA family and the species. Traditionally, satDNAs were considered to exist in large arrays, often localised in centromeric or subtelomeric regions of chromosomes (Mlinarec et al. 2019; Orzechowska et al. 2018; Ribeiro et al. 2020; Yücel et al. 2024). In *C. capillaris* genome, all analysed repeat families formed large arrays that were readily detectable using FISH, as it was shown earlier by Jamilena et al. (1993). However, recent bioinformatic analyses of long-read sequencing data from both plant and animal models have revealed that many satDNA families form arrays of varying lengths, with the majority being relatively short and only a few forming long arrays (Larracuente 2014; Ruiz-Ruano et al. 2016; Vondrak et al. 2020). A similar pattern was observed for the pCcD29 repeat, in *C. taraxacifolia*, both long and short arrays were present, whereas in *C. polymorpha*, the arrays were relatively short but still detectable by FISH. It is also possible that short arrays of this repeat exist in the *C. capillaris* genome alongside the large arrays; however, their length may be insufficient for FISH detection. In *C. vesicaria*, pCcD29 repeat was, according to the Southern blot, relatively abundant but unable to be detected with FISH, suggesting the organisation in short arrays below the detection threshold of FISH. This observation is consistent with previous studies showing that the same satellite repeat can form long arrays in one species but only short arrays in a closely related species (Camacho et al. 2022; Yücel et al. 2024).

Ruiz-Ruano et al. (2016) proposed a model of evolution of satDNAs (Fig. 4c) containing three main steps: (i) de novo duplication of sequence, e.g., via unequal crossing over or polymerase slippage (the rise of new tandem repeat); (ii) dissemination of the new short arrays throughout the genome by, e.g., transposable elements or extrachromosomal circular DNA; and finally (iii) one or few of the short arrays undergo amplification and create large arrays. The chromosomal distribution of pCcD29 repeat observed in analysed *Crepis* species might depict steps of the model from the appearance of multiple short repeats (*C. polymorpha*) through diversification of longer arrays (*C. taraxacifolia*) to a few major loci consisting of long arrays of repeats (*C. capillaris*; Fig. 4c). This observation is consistent with several earlier studies, which demonstrated that the molecular drive leading to the concerted evolution pattern of satDNA is a time-dependent process (e.g., Bachmann and Sperlich 1993; Pérez-Gutiérrez et al. 2012; Strachan et al. 1985; Ugarković and Plohl 2002).

The obtained results suggest that the three repeats were present in the genome of a common ancestor of subclade IVc, after which each followed a distinct evolutionary trajectory (Fig. 4b). Analyses of the species distribution of the satDNAs within a phylogenetic framework indicate several events of satDNA amplification (Fig. 4b). The amplification of the pCcD29 repeat seems to have occurred along the same evolutionary branches as descending dysploidy events associated with the speciation of *C. capillaris* (a reduction in chromosome number from *x* = 4 to *x* = 3) and the evolution of a common ancestor of *C. vesicaria* group (Senderowicz et al. 2021). The genomic organisation of the pCcD29 repeat is very similar within the *C. vesicaria* group. However, FISH results suggest that speciation within this group was accompanied by diversification in the chromosomal distribution of this repeat. Similar results were earlier reported for *Lagoseris* lineage of *Crepis s.l*. and *Lathyrus* species (Ceccarelli et al. 2010; Yücel et al. 2024). Interestingly, while the pCcD29 sequence family appears to be homogenised in *C. capillaris*, within the *C. vesicaria* group, three distinct subfamilies were amplified. It remains unclear whether this lower level of homogenisation is related to the higher number of loci composed of short arrays, as intralocus homogenisation is considered more efficient than interlocus one (Matyasek et al. 2011; Pinhal et al. 2011; Yang et al. 2020).

The satDNA family pCcH32 seems to be present in species from subclade IVc and possibly also subclade IVb, since Southern hybridisation with lower stringency showed its moderate amplification in *C. syriaca* and *C. alpina*. The high amplification of this repeat was revealed only for *C. capillaris* and to a lesser extent in *C. oporinoides*. It should be added that hybridisation signals for other species than *C. capillaris* were obtained only in the lower stringency experiment, indicating that other more divergent subfamilies than the one isolated from *C. capillaris* were present in their genomes. It cannot be excluded that repeat sequences with sequence identity less than 72% were present in other species. The accumulation of mutations contributes to the emergence of new satDNA variants, which can subsequently spread within the genome and potentially replace older ones (birth-and-death model of molecular evolution; Garrido-Ramos 2017; Subirana et al. 2015).

The amplification of the studied satDNA families seems to be associated with dysploidy events in the analysed *Crepis* species. The dysploidy event (from *x* = 4 to *x* = 3) during the speciation of *C. capillaris* was accompanied by the amplification of all three examined satDNAs. The origin of the common ancestor of the *C. vesicaria* group was associated with independent amplification events of pCcD29 and a reduction in chromosome number from *x* = 5 to *x* = 4 (Fig. 4b). In contrast, although the amplification or elimination of pCcH32 seems to have accompanied the speciation of several of the studied species, our results do not support an association between dysploidy events and pCcH32 evolution (except for the speciation of *C. capillaris*; Fig. 4b). Repetitive sequences are considered to be hotspots of chromosomal recombination. Long arrays of identical or nearly identical repeats are particularly prone to recombination events, which can lead to large-scale chromosomal rearrangements such as Robertsonian fusions or translocations, the mechanisms underlying dysploidy (Golczyk et al. 2022; Louzada et al. 2020; Molnár et al. 2011).

## Conclusion

Despite sharing the same repeat library, each satDNA family evolves independently in *Crepis* species. Amplification of pCcD29 and pCcE9 seems to accompany the dysploidy events. High amplification of pCcE9 was characteristic only of *C. capillaris*. Although pCcH32 was present in many of the studied species, the techniques used to assess its genomic abundance showed that it is highly amplified only in *C. capillaris* and *C. oporinoides*. Different evolutionary patterns were revealed for the third analysed satDNA family, pCcD29, which was not only present in nearly all species from subclade IVc, but was also highly amplified in some of them (the *C. vesicaria* group). Interestingly, the genomic organisation of pCcD29 differed from that in *C. capillaris*, which may result from lower homogeneity of this repeat in the *C. vesicaria* group. While the species from the *C. vesicaria* group share the same genomic organisation, their chromosomal distribution patterns differed significantly. The observed patterns of chromosomal distribution support the hypothesis of satellite DNA evolution proposed for animal models by Ruiz-Ruano et al. (2016). Distinct chromosomal and genomic organisation patterns of the same satDNA family (pCcD29) were observed among the analysed *Crepis* species, ranging from numerous small loci and low degree homogenisation (*C. polymorpha*) to a few major loci exhibiting a high degree of homogenisation (*C. capillaris*), which colocalised with large heterochromatin blocks in *C. capillaris* chromosomes as was shown by Jamilena et al. (1993). Such heterochromatic blocks are considered to play important roles in maintaining genome integrity, proper gene regulation, and nuclear organisation (De Storme and Mason 2014; Garrido-Ramos 2017; Janssen et al. 2018; Louzada et al. 2020; Wang et al. 2024).

## Supplementary Information

Below is the link to the electronic supplementary material.

Supplementary file1 (DOCX 4277 KB)

## Data Availability

Supplementary data are available at *Chromosome Research* online. Sequence data that support the findings of this study have been deposited in the NCBI with the primary accession codes: PV520719-PV520723; PV520747-PV520756; PV520767-PV520771; PV520780-PV520788; PV520795-PV520811; PV520843-PV520858; PV520886-PV520899; PV520905-PV520908.
