# Supplementary material for: From minor loci to major players? Satellite DNA diversification in Crepis sensu stricto
Source: Chromosome Res. 2025 Nov 5;33(1):25. doi: 10.1007/s10577-025-09783-1 (PMC12589258; doi:10.1007/s10577-025-09783-1)
Supplement: Supplementary file 1 — Supplementary file1 (DOCX 4277 KB) [file 10577_2025_9783_MOESM1_ESM.docx]

**Supplementary Information**

**From minor loci to major players? Satellite DNA diversification in *Crepis sensu stricto.***

**Magdalena Senderowicz^1^, Natalia Borowska-Żuchowska^1^, Gülru Yücel^1,2^, Teresa Nowak^1^ Gbemisola Daini^1^ and Bożena Kolano^1^**

^1^Plant Cytogenetics and Molecular Biology Group, Institute of Biology, Biotechnology and Environmental Protection, Faculty of Natural Sciences, University of Silesia in Katowice, Katowice 40-032, Poland

^2^Faculty of Agriculture, Department of Agricultural Biotechnology, Ondokuz Mayıs University, Samsun 55200, Türkiye

*Correspondence should be addressed to Bozena Kolano, 28 Jagiellonska Street, Katowice 40-007, Poland Tel: +482009468; Email: [bozena.kolano@us.edu.pl](mailto:bozena.kolano@us.edu.pl)

**Supplementary Table 1**

PCR amplification and sequencing information

| Region | MgCl2 concentration | Annealing temperature | Primer name and sequence (5’-3’) |
| --- | --- | --- | --- |
| pCcE9 | 2.5 mmol/L | 52 °C | Forward: TTTTGAGCTGTCGAGTGCCA  Reverse: CGGTGCAACTTTTCCCGATC |
| pCcD28 | 2.5 mmol/L | 52 °C | Forward: TTCAAGGATCGATTGGACGGC  Reverse: GGAAGGAGAACAACGTCTAAAGG |
| pCcH32 | 3 mmol/L | 55 °C | Forward: GTTGTCATTCAAACCCTAGTTCG  Reverse: GGTTGGAACTTCGGGATGG |

**
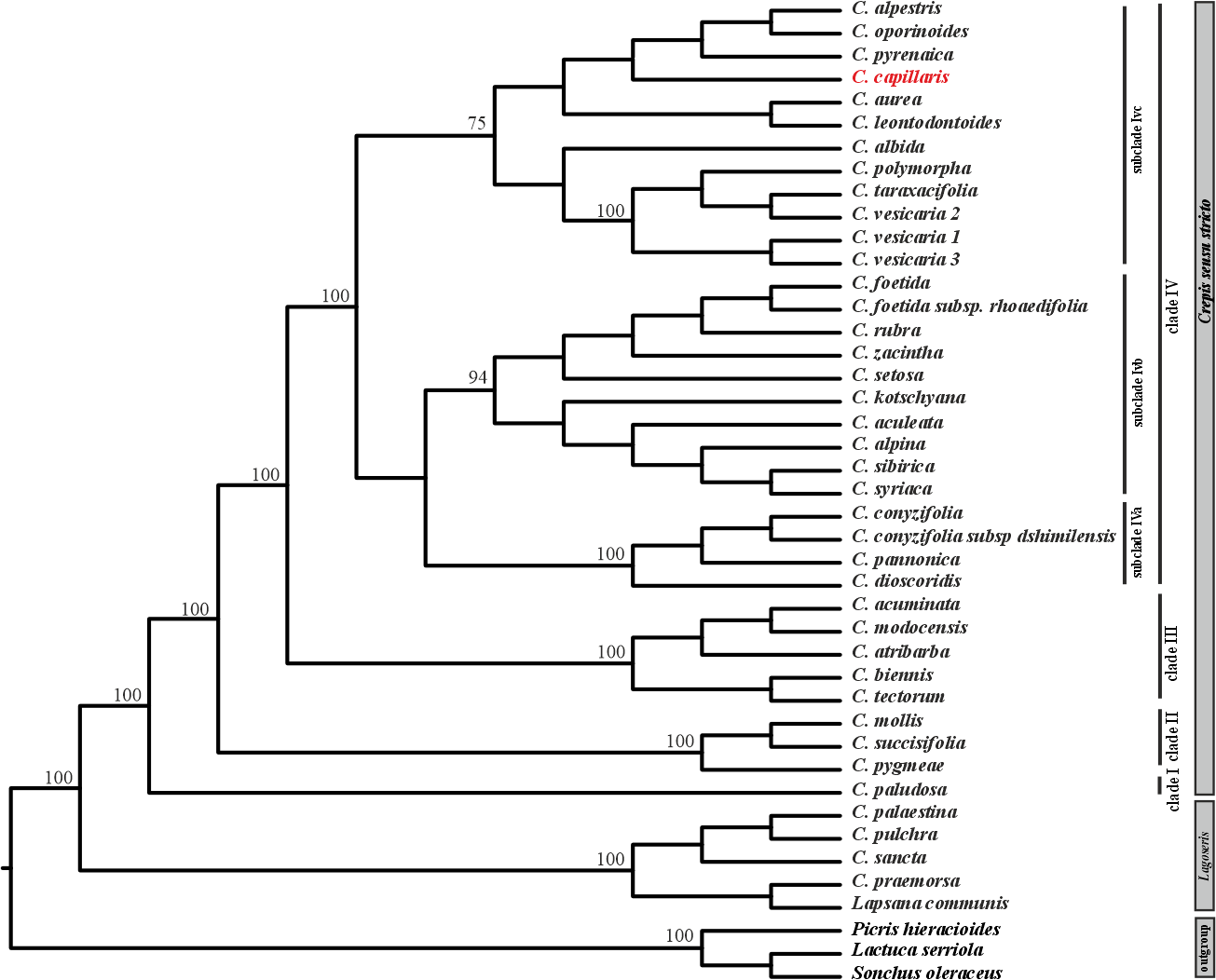
Supplementary Fig. 1**

Phylogenetic relationships among the analysed *Crepis* species based on the cpDNA data sets obtained in Senderowicz et al., 2021. Bootstrap support values are indicated at each node. The tree was rooted with *Picris hieracioides*, *Lactuca serriola*, and *Sonchus oleraceus*


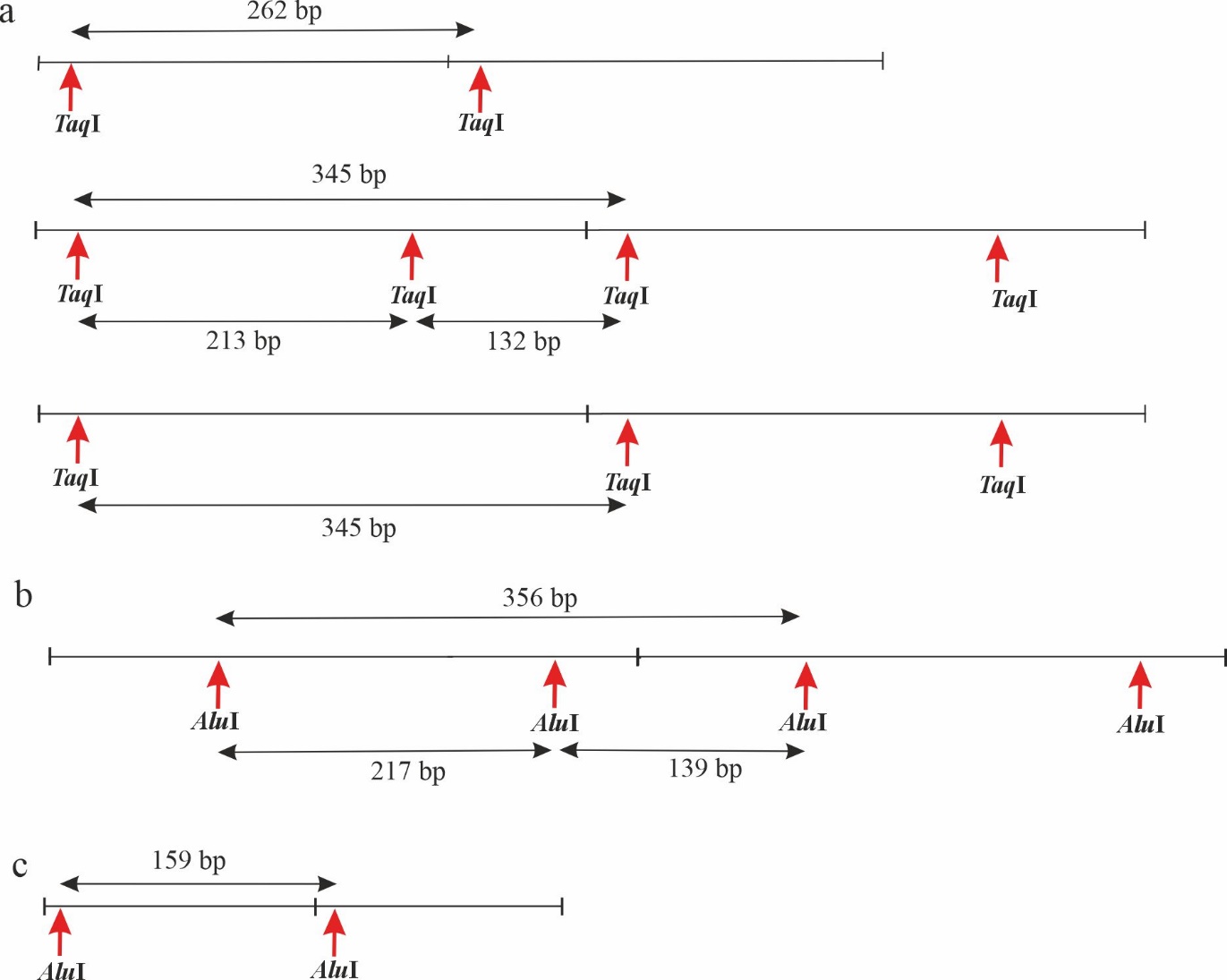


**Supplementary Fig. 2**

Most frequent restriction enzyme cleavage sites according to cloned sequence analysis. (A) pCcD29; One or two *Taq*I cleavage sites were present, depending on the variant. The monomer of 262 bp long represents sequences from cluster 1, while the monomer 345 bp long represents sequences from cluster 2 (Fig. 1D). (B) pCcE9 (C) pCcH32. Red arrows indicate sites recognised by the restriction enzymes (*Alu*I or *Taq*I). The bars represent the lengths of the monomers after restriction

**
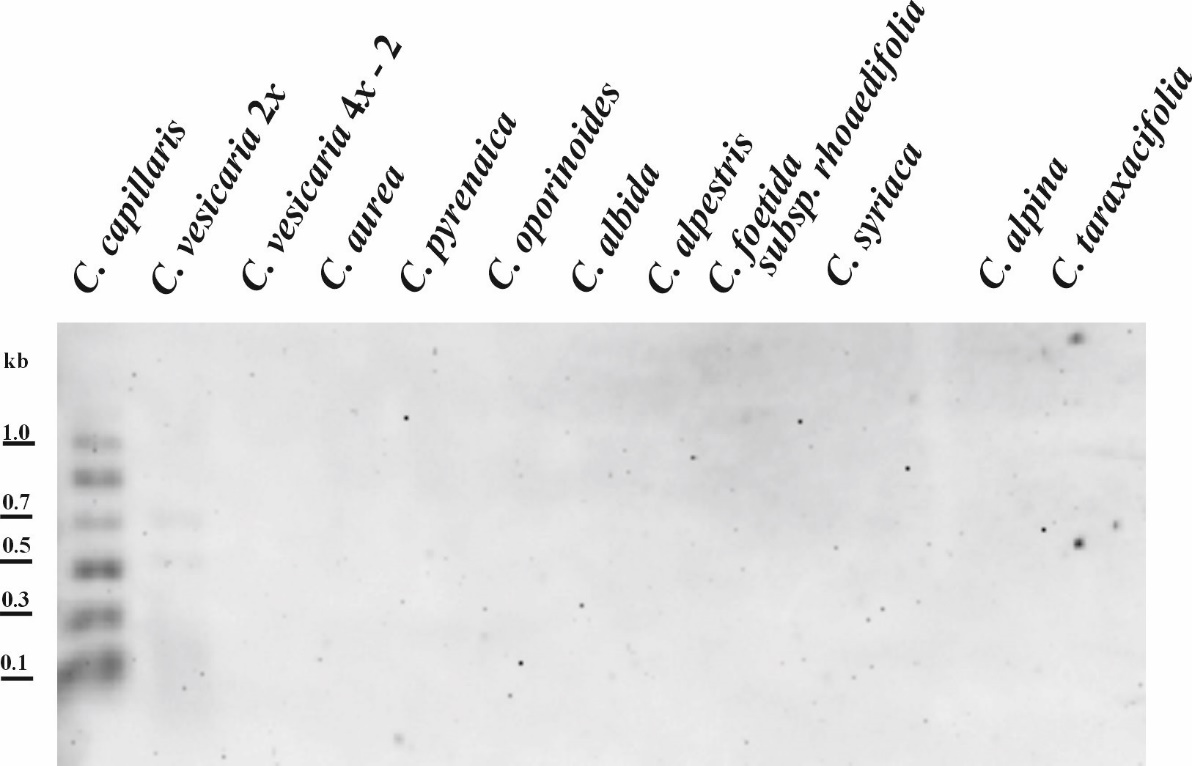
**

**Supplementary Fig. 3**

Southern hybridisation of cloned pCcH32 satDNA to genomic DNA of analysed species restricted with *Alu*I (stringency 81 %)
